# Supplementary material for: Growth Hormone Upregulates Melanocyte-Inducing Transcription Factor Expression and Activity via JAK2-STAT5 and SRC Signaling in GH Receptor-Positive Human Melanoma
Source: Cancers (Basel). 2019 Sep 12;11(9):1352. doi: 10.3390/cancers11091352 (PMC6769493; doi:10.3390/cancers11091352)
Supplement: Supplementary file 1 [file cancers-11-01352-s001.pdf]

Title: **Growth hormone (GH) upregulates melanocyte inducing transcription factor (MITF) expression and activity via JAK2-STAT5 and SRC signaling in GH receptor (GHR) – positive human melanoma.**

Authors: Reetobrata Basu[1], Prateek Kulkarni[1,3], Yanrong Qian[1], Christopher Walsh[4], Pranay Arora[4], Emily Davis[1,2], Silvana Duran-Ortiz[1,3], Kevin Funk[1,3], Diego Ibarra[1,2], Colin Kruse[1,3], Sam Mathes[1], Todd McHugh[1], Alison Brittain[3,4], Darlene E. Berryman[1,4,5], Edward O. List[1], Shigeru Okada[1,4], John J. Kopchick[1,4]

Affiliations: *[1]Edison Biotechnology Institute, Ohio University (OU), [2]Dept. of Chemistry and Biochemistry, OU, [3]Molecular and Cellular Biology (MCB) Program, Dept. of Biological Sciences, OU, [4]Dept. of Biomedical Sciences, Ohio University Heritage College of Osteopathic Medicine, Athens, OH, [5]The Diabetes Institute, Ohio University, Athens, OH*

SUPPLEMENTARY FIGURES

Sup-Fig 1: Drug treatment induced autocrine GH expression correlates with ABC-transporter expression in human melanoma cells → DOX = Doxorubicin; VEM = Vemurafenib; CRI = Crizotinib; CAB = Cabozantinib

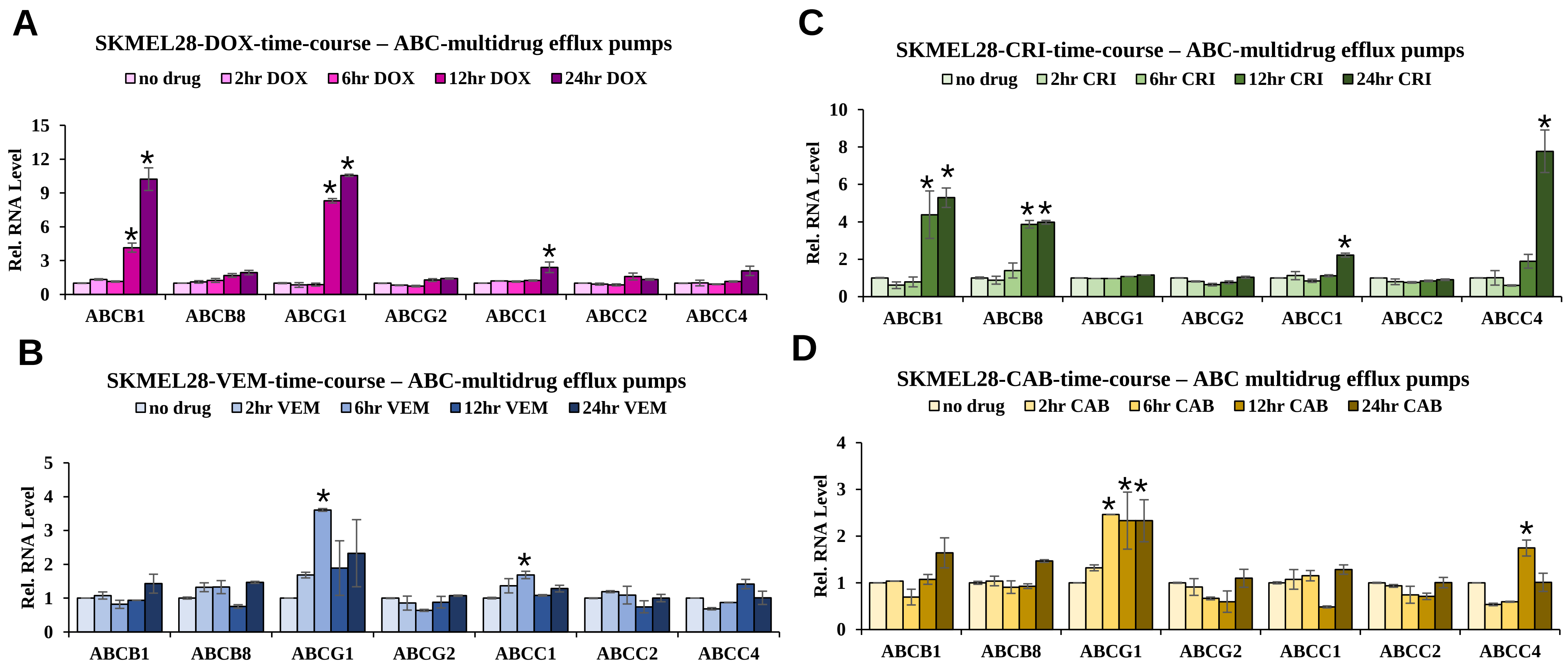

Sup-Fig 1: Drug treatment induced autocrine GH expression correlates with ABC-transporter expression in human melanoma cells: (A-D) – Human melanoma cells SK-MEL-28 were treated with anti-cancer compounds and RNA expressions was compared with respective untreated controls, at 2, 6, 12, and 24-hr timepoints by RT-qPCR with corresponding pre-validated primers (sequence in supplementary table 1) for target genes. Changes in GH are shown in Fig-1. Here changes in expression of ABC-transporters with time, to doxorubicin (A), vemurafenib (B), crizotinib (C) and cabozantinib (D) treatments are shown. RNA expressions were quantified by RT-qPCR and normalized against expression of bTUB and ACTB as reference genes [*, $p < 0.05$, Wilcoxon sign rank test, $n = 3$].

Sup-Fig 1E: GH treatment directly upregulates ABC-transporter gene expression in human melanoma cell SK-MEL-28

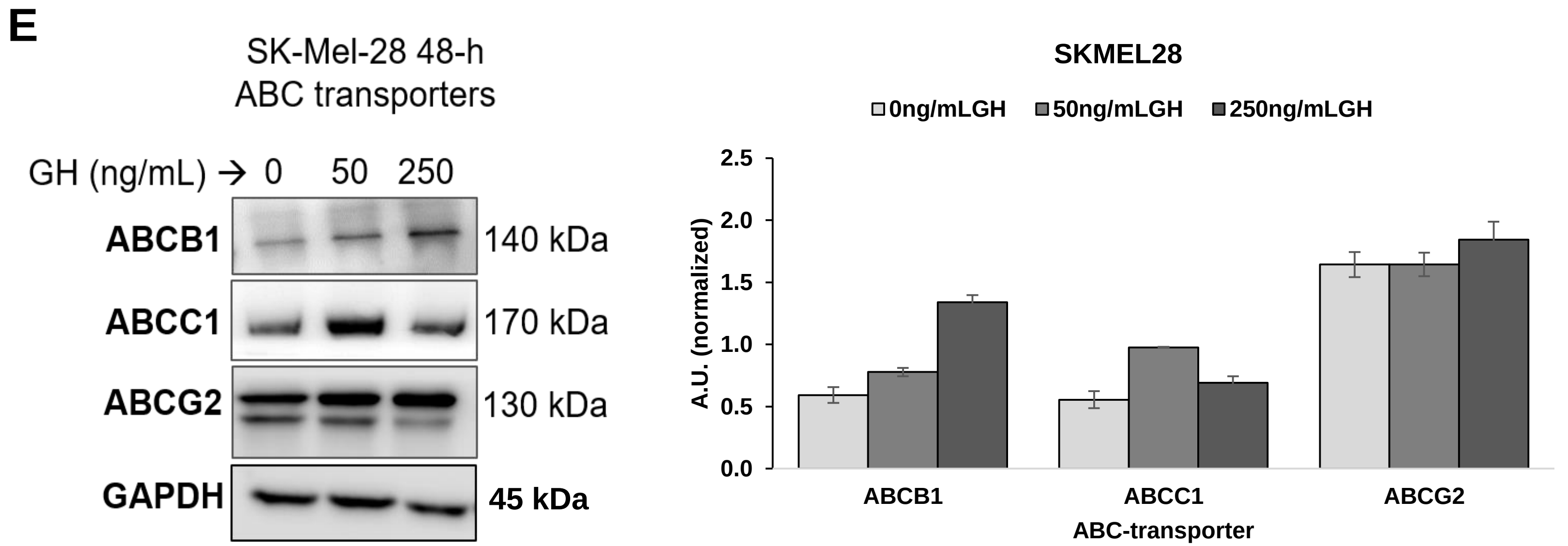

Sup-Fig 1E: GH treatment directly upregulates ABC-transporter proteins in human melanoma cells: F – Human amelanotic melanoma cells SK-MEL-28 were treated with increasing doses (0, 50, 250ng/mL) of recombinant human growth hormone (GH) for 48-hr and cell lysate was analyzed by western blot for ABC-transporters ABCB1, ABCC1, and ABCG2. Image quantification was done using ImageJ and results were normalized against GAPDH expression.

Sup-Fig 2: GH treatment directly upregulates MITF and MITF target RNA expressions in human melanoma cells: (A-B) – Human melanotic melanoma cells SK-MEL-30 were treated with increasing doses (0, 50, 100, 200ng/mL) of recombinant human growth hormone (GH) and heatmap showing changes in RNA expressions at 6, 24, and 48-hr timepoints were analyzed for GH, GHR, MITF and a number of MITF-targets, as well as ABC-transporters ABCB5, ABCG2 (A); Identical experiment was performed in presence of 200nM doxorubicin (B). Numbers inside boxes indicate fold-change in gene expression compared to GH untreated control. Similar set of experiments for amelanotic melanoma cells SK-MEL-28 is shown in Sup-Fig 2. Further, using melanotic melanoma cells MALME-3M **(C)** and MDA-MB-435 **(D)**, we treated with either doxorubicin (dox), or cisplatin (cis), or vemurafenib (vem) for 24-hr timepoint only, in absence / presence of 50ng/mL GH along with siRNA-mediated GHRKD. Drug-specific response was seen, and GHRKD suppressed expression of multiple MITF-targets. Similar experiments with MDA-MB-435 cells is shown in Sup-Fig 3. RNA expressions were quantified by RT-qPCR and normalized against expression of βTUB and ACTB as reference genes [*, $p < 0.05$, Wilcoxon sign rank test, $n = 3$].

Sup-Fig 2: GH treatment directly upregulates MITF and MITF target RNA expressions in human melanoma cells

A

SKMEL30	6-h				24-h				48-h			
	0 GH	50 GH	100 GH	200 GH	0 GH	50 GH	100 GH	200 GH	0 GH	50 GH	100 GH	200 GH
GH1	1.0	1.0	0.4	0.5	1.0	0.9	0.6	0.6	1.2	1.2	1.1	1.1
GHR	1.0	1.1	1.3	0.7	0.9	1.1	0.5	1.5	1.0	0.9	1.2	0.8
MITF	1.0	1.1	1.3	1.2	0.8	0.8	0.8	1.0	0.5	0.5	0.6	0.7
PGC1a	1.0	1.2	1.4	1.4	0.8	0.9	0.9	1.2	0.6	0.7	1.0	0.9
PMEL	1.0	1.0	0.9	1.2	1.0	0.7	0.9	1.0	1.2	1.0	0.7	1.4
UCP1	1.0	1.1	1.5	0.9	1.0	1.3	1.2	1.1	1.3	1.2	1.0	1.1
TYR	1.0	1.1	0.8	0.5	1.0	0.8	0.7	0.9	0.6	0.5	0.6	0.8
TYRP1	1.0	0.9	1.0	0.9	0.8	0.9	0.8	1.0	0.7	0.6	0.6	0.8
MLANA	1.0	0.7	1.0	1.0	1.4	1.2	1.3	2.1	1.0	1.2	0.7	0.9
ABCB5	1.0	1.4	1.4	1.3	1.9	1.7	1.8	2.1	0.9	1.2	1.3	1.2
ABCG2	1.0	0.8	0.7	0.8	0.8	0.7	0.8	1.0	1.0	0.9	1.0	0.8
HIF1a	1.0	1.3	1.0	0.7	0.8	0.7	0.7	0.9	1.2	1.1	1.1	1.2
BCL2	1.0	1.6	1.4	1.1	1.6	1.9	1.5	1.5	1.0	0.9	1.3	0.8
BRCA1	1.0	1.0	0.8	1.1	2.9	2.8	2.2	2.5	2.1	1.4	1.2	1.2
OCT	1.0	0.9	1.0	1.2	1.6	1.3	1.2	1.4	1.2	1.0	0.9	1.1
MET	1.0	0.8	1.0	1.0	0.7	0.7	0.7	0.9	0.6	0.5	0.6	0.7
CDKN1A	1.0	0.7	1.5	1.1	0.8	1.2	0.9	1.5	1.7	1.8	1.7	1.9

B

SKMEL30	6-h				24-h				48-h			
	0GH doz	50GH doz	100GH doz	200GH doz	0GH doz	50GH doz	100GH doz	200GH doz	0GH doz	50GH doz	100GH doz	200GH doz
GH1	1.0	1.0	0.7	1.0	1.0	1.7	1.5	1.4	1.0	1.4	1.2	1.4
GHR	1.0	1.5	1.2	1.4	1.0	1.2	1.6	2.1	1.0	2.2	2.0	2.3
MITF	1.0	1.1	0.9	1.7	1.0	0.9	0.9	1.1	1.0	1.0	1.0	1.3
PGC1a	1.0	1.0	0.8	1.3	1.0	1.1	1.1	2.1	1.0	1.1	1.0	1.4
PMEL	1.0	2.0	2.1	2.0	1.0	1.1	1.1	1.4	1.0	1.3	1.2	1.3
UCP1	1.0	1.0	0.9	0.8	1.0	1.3	1.2	1.1	1.0	1.2	1.3	1.1
TYR	1.0	1.1	1.3	1.7	1.0	0.9	0.9	1.2	1.0	0.7	0.8	1.3
TYRP1	1.0	1.0	1.3	1.6	1.0	0.9	1.1	1.2	1.0	1.4	1.2	1.5
MLANA	1.0	1.6	1.9	4.1	1.0	1.2	1.6	2.1	1.0	1.1	1.8	2.4
ABCB5	1.0	1.8	1.8	1.9	1.0	2.2	2.0	1.7	1.0	1.7	2.2	2.5
ABCG2	1.0	0.9	0.7	0.8	1.0	1.0	0.9	1.0	1.0	1.2	1.0	1.2
HIF1a	1.0	1.2	1.2	1.4	1.0	0.7	1.0	1.4	1.0	1.2	0.9	1.0
BCL2	1.0	1.0	1.0	1.3	1.0	1.3	1.1	2.0	1.0	1.3	0.8	1.1
BRCA1	1.0	0.8	1.2	1.7	1.0	0.9	1.2	1.4	1.0	1.2	1.0	1.6
OCT	1.0	1.2	0.8	1.4	1.0	0.6	0.9	1.0	1.0	1.0	0.6	0.8
MET	1.0	1.4	1.1	1.2	1.0	1.1	1.2	1.5	1.0	1.2	0.8	1.0
CDKN1A	1.0	0.7	0.3	0.5	1.0	2.0	1.3	1.4	1.0	1.1	0.8	1.4

FOLD INCREASE

10.0
5.0
2.5
1.3
1.0
0.8
0.4
0.2
0.1

FOLD DECREASE

Sup-Fig 2: GH treatment directly upregulates MITF and MITF target RNA expressions in human melanoma cells

C

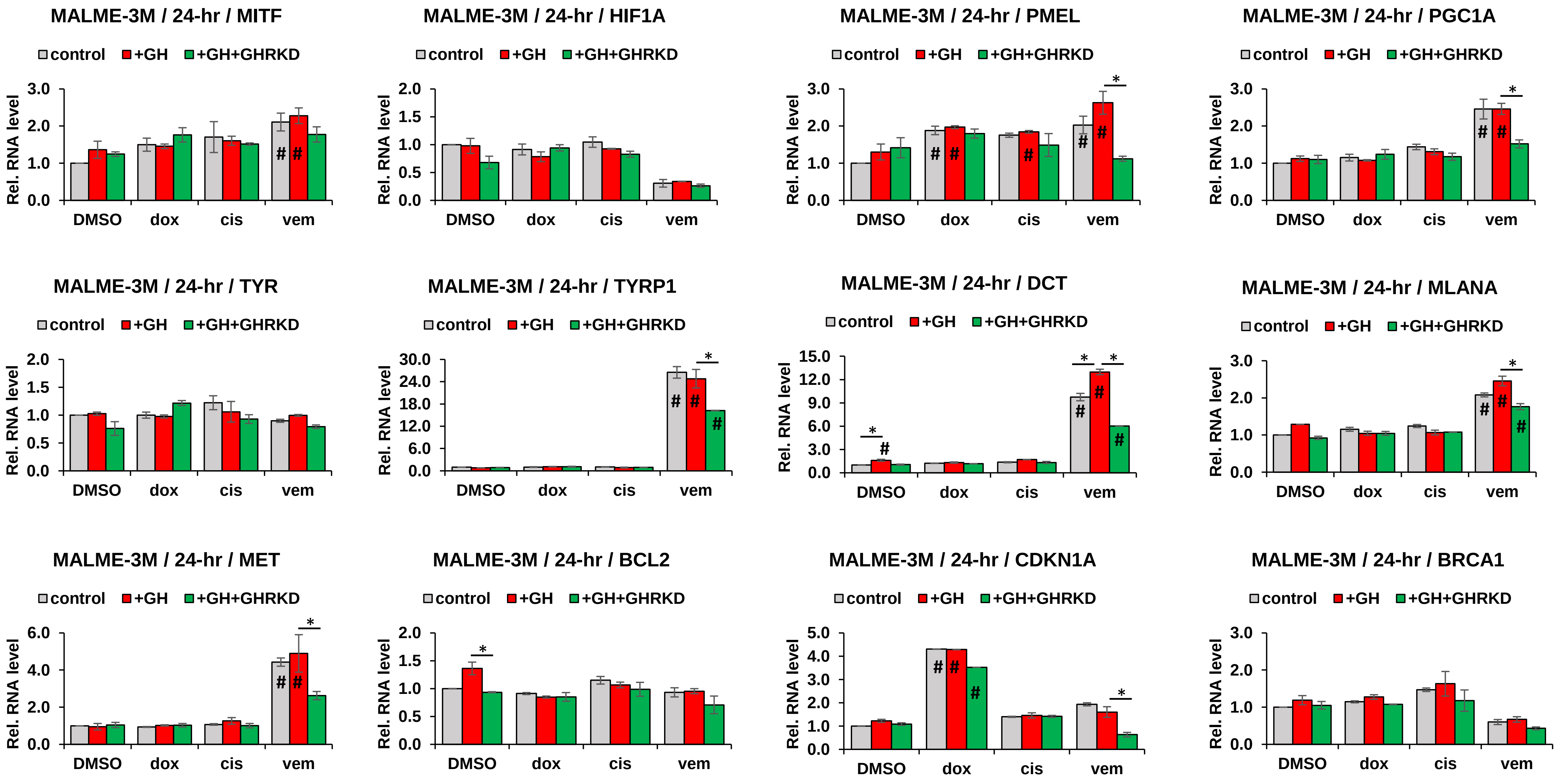

Sup-Fig 2: GH treatment directly upregulates MITF and MITF target gene expressions in human melanoma cells → MDA-MB-435

D

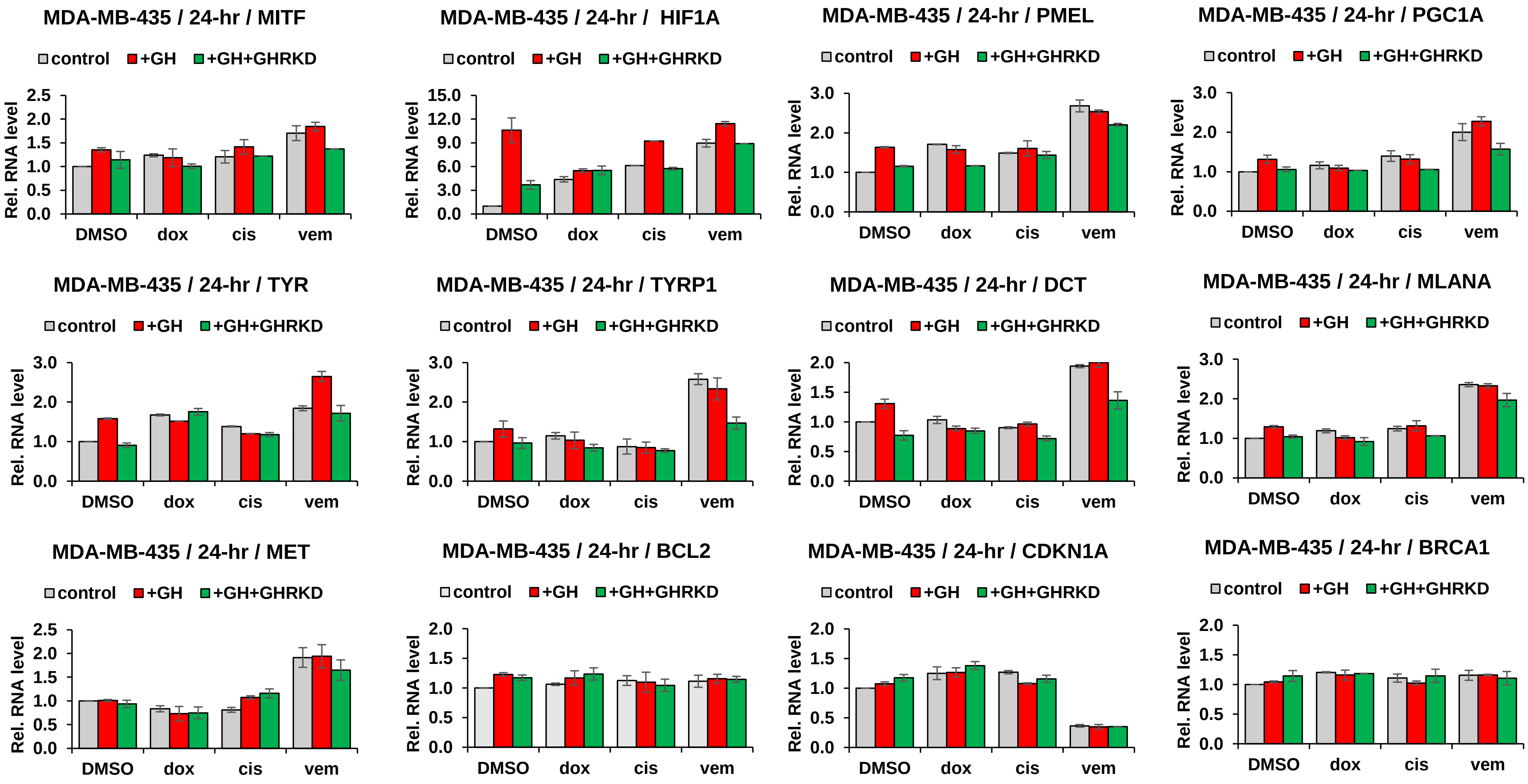

Sup-Fig 3: Effect of bovine growth hormone treatment on B16F10 mouse melanoma cells

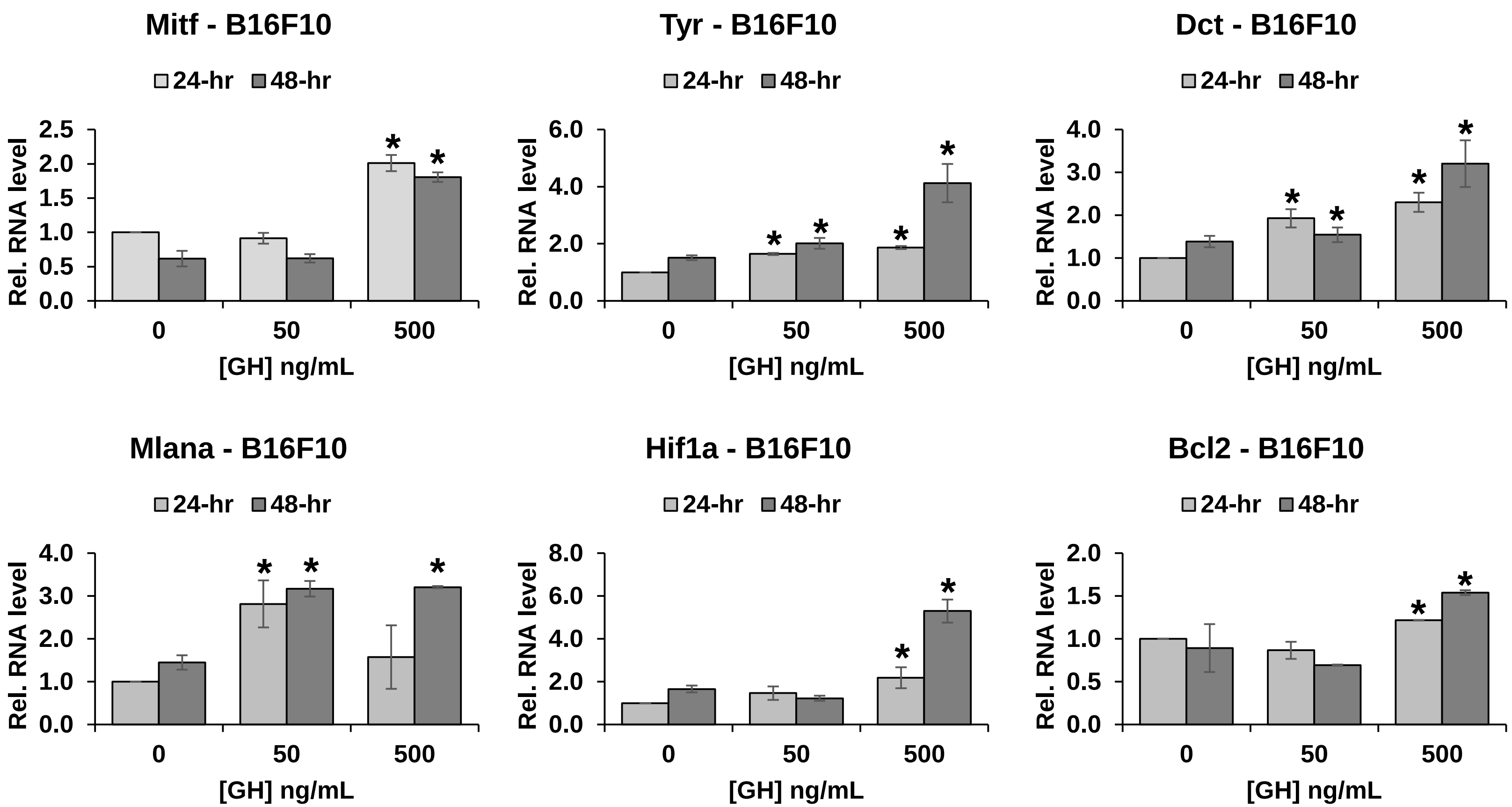

Sup-Fig 3: Effect of bovine growth hormone treatment on B16F10 mouse melanoma cells: Mouse melanotic melanoma cells B16F10 were treated with increasing doses (0, 50, 500ng/mL) of recombinant bovine growth hormone (bGH) for 24, and 48-hr timepoints and expression levels of Mitf and Mitf target genes were analyzed. RNA expressions were quantified by RT-qPCR and normalized against expression of βTUB and ACTB as reference genes [*, $p < 0.05$, Wilcoxon sign rank test, n = 3].

Sup-Fig 4: GH induced MITF increases PGC1A level and activity differentially

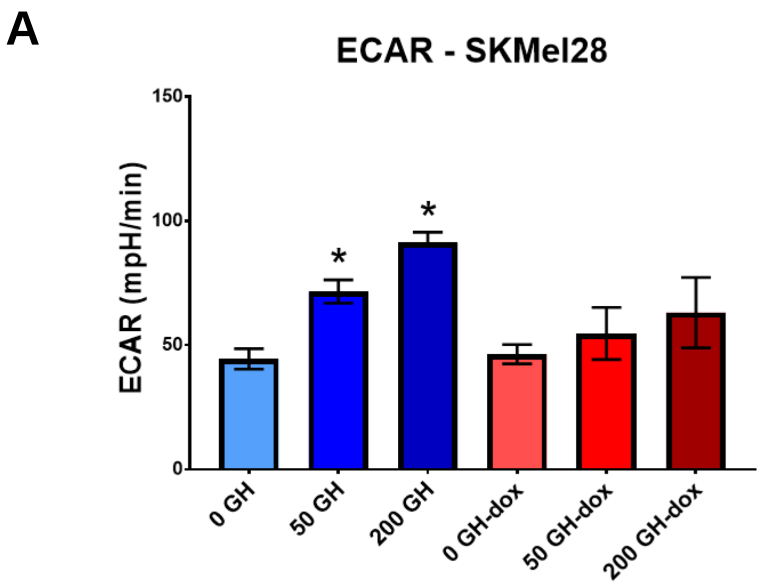

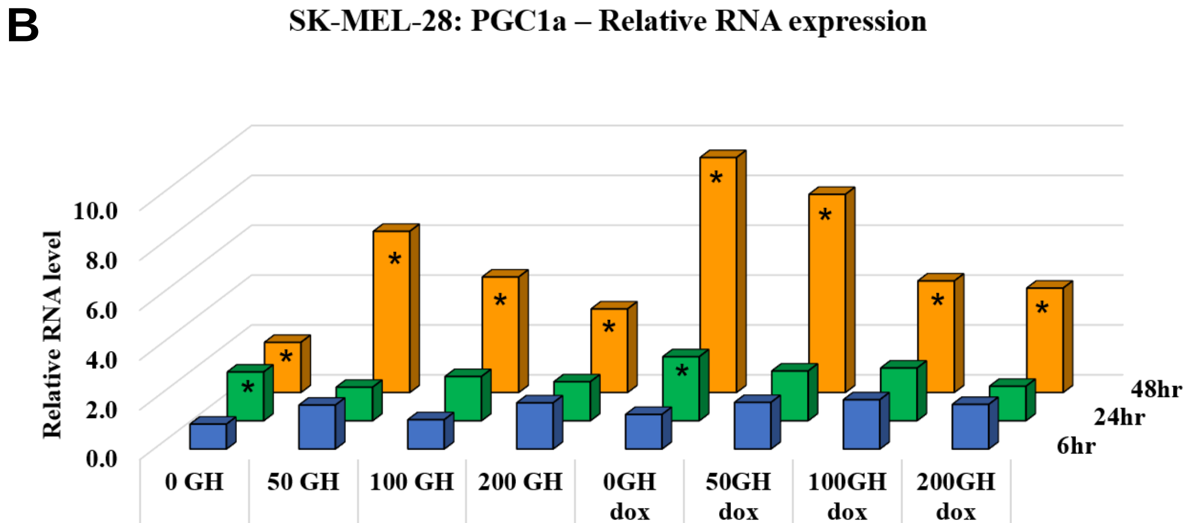

	0 GH	50 GH	100 GH	200 GH	0GH dox	50GH dox	100GH dox	200GH dox
6hr	1.0	1.8	1.2	1.9	1.4	1.9	2.0	1.8
24hr	2.0	1.3	1.8	1.6	2.6	2.0	2.1	1.4
48hr	2.0	6.5	4.6	3.4	9.4	7.9	4.5	4.2

Sup-Fig 4: GH enhances MITF target PGC1A expression and activity in human melanoma: Human melanoma SK-MEL-28 cells show GH dose dependent (0, 50, 200ng/mL) increase in glycolysis (ECAR, extracellular acidification rate) in absence of chemotherapy. In presence of chemotherapy (200nM doxorubicin) the glycolytic increase was suppressed. (B) Mitochondrial OX-PHOS regulator PGC1A RNA expressions +/- GH, +/-doxorubicin treatments were quantified by RT-qPCR and normalized against expression of bTUB and ACTB as reference genes [*, $p < 0.05$, Wilcoxon sign rank test, $n = 3$].

Sup-Fig 5: bGH mice melanoma tumors have higher endogenous GH production compared to WT mice tumors

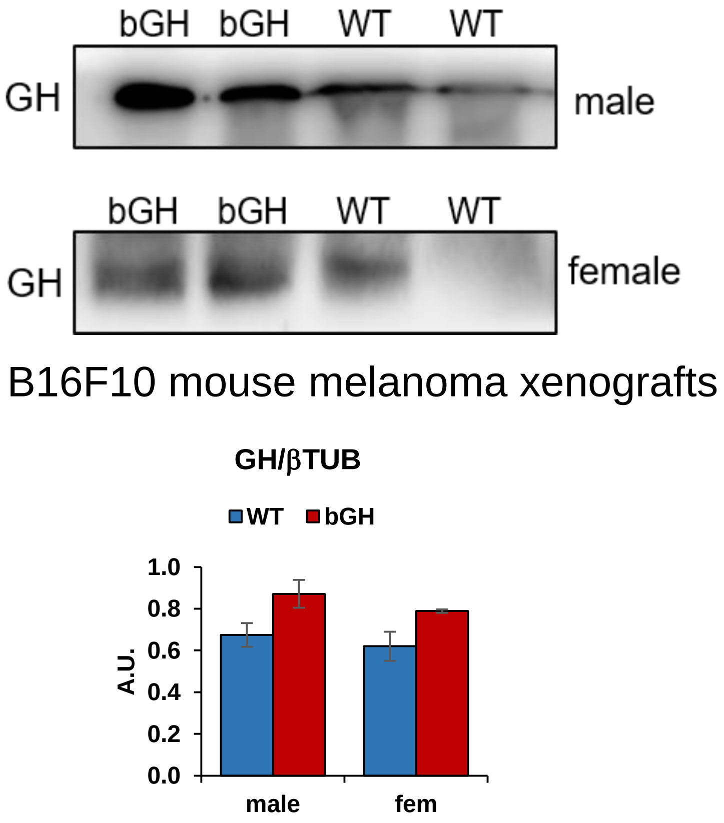

Sup-Fig 6: Melanoma stage specific deaths in GHR-high and GHR-low male and female melanoma patients in TCGA dataset

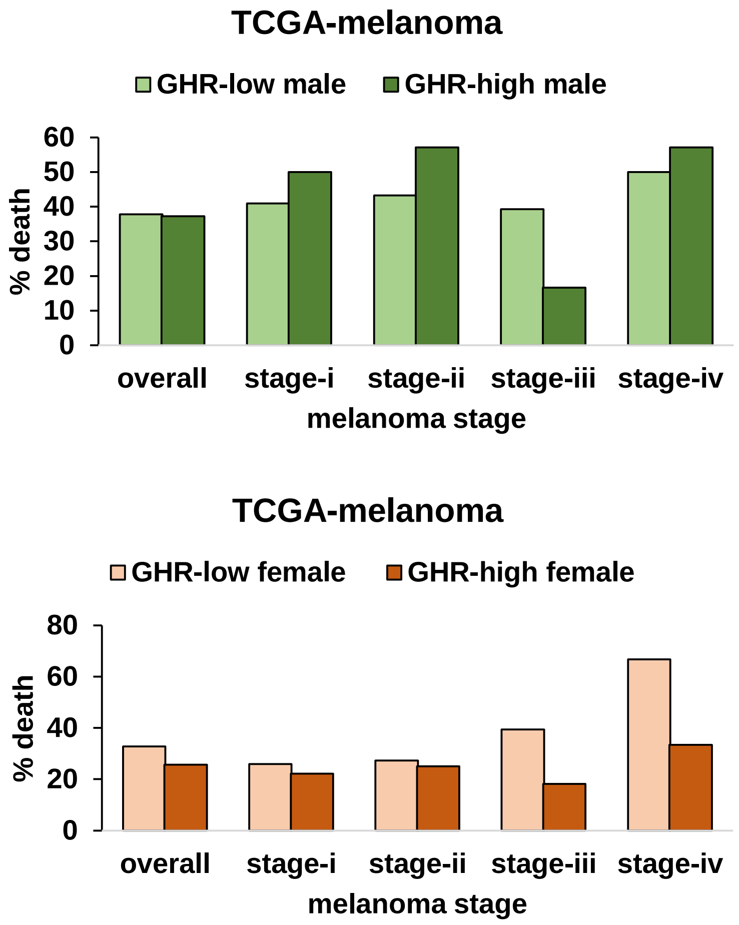

Sup-Fig 7: Bioinformatic analysis: GHR, MITF, and MITF target genes co-express and strongly cluster (green box) in the CCLE dataset

Cancer Cell Line Encyclopedia (CCLE); 967 samples

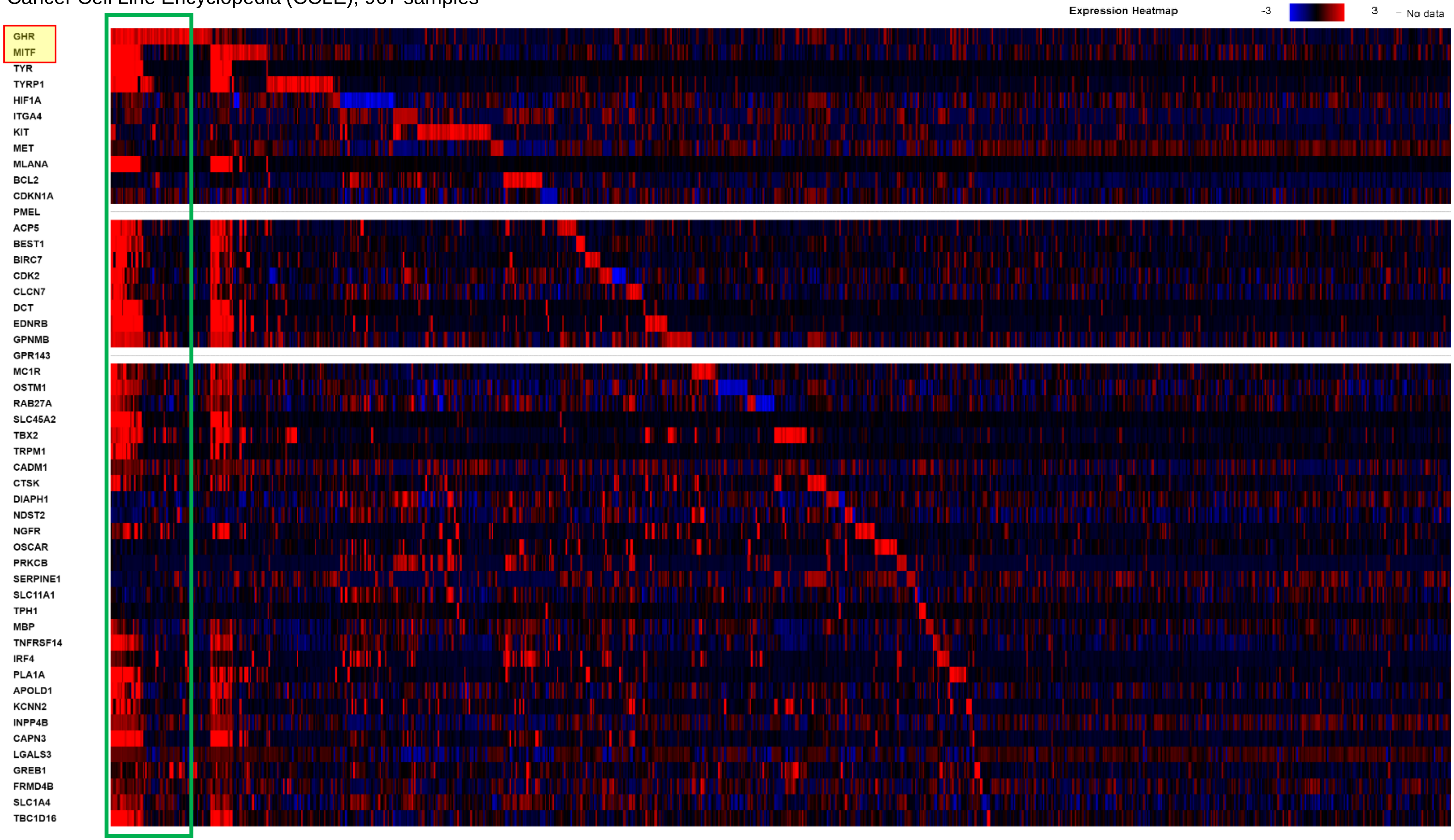

Sup-Fig 8: Bioinformatic analysis: TCGA datasets analyzed in the context of high (above mean) and low (below mean) GHR expressions show upregulated MITF, MITF targets, and ABC transporter expressions in the GHR-high cohort in both males and females

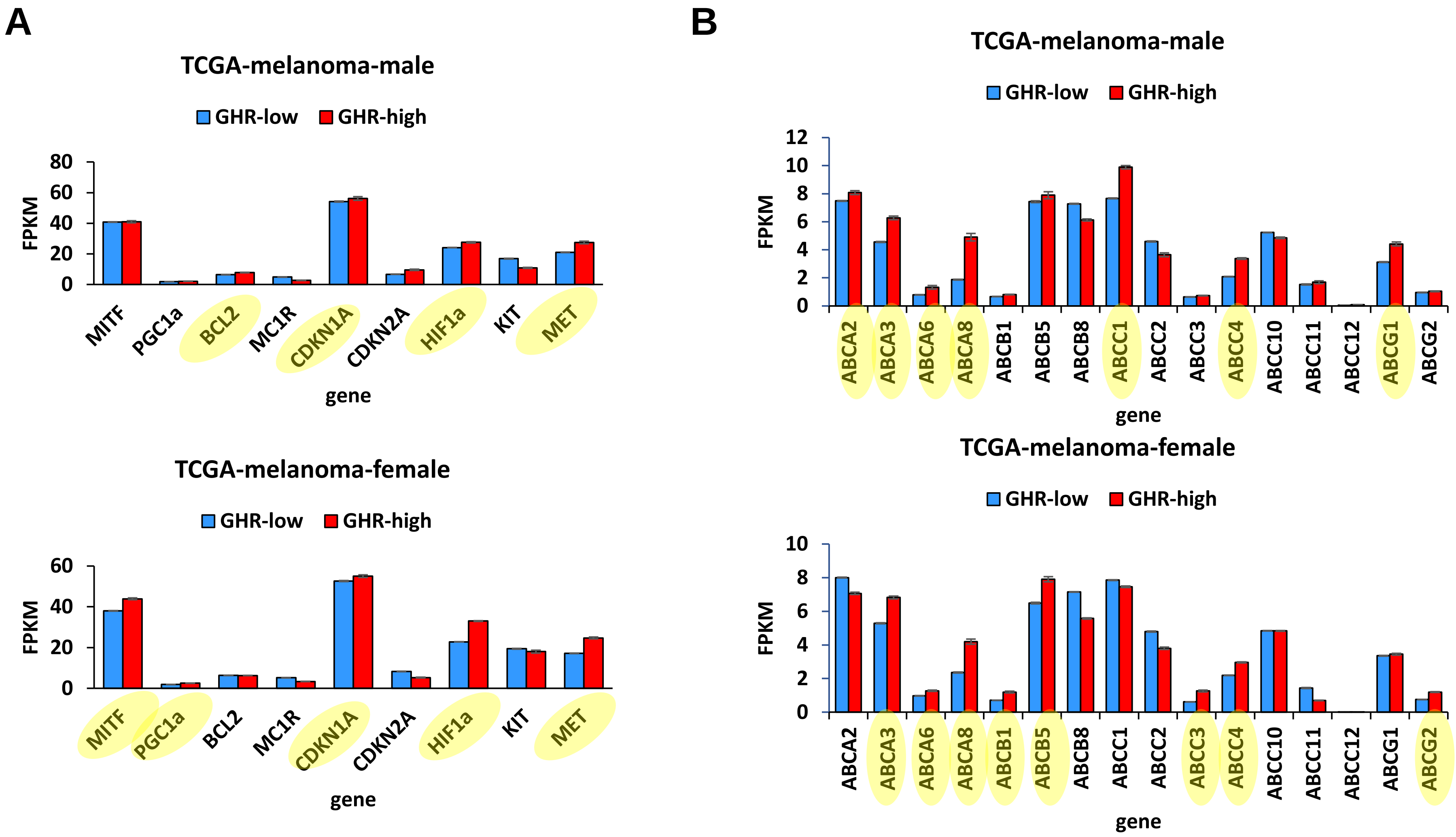

Sup-Fig 9: GH regulated MITF and MITF target gene regulation proceeds via JAK2-STAT5 and SRC regulated pathways → SK-MEL-28

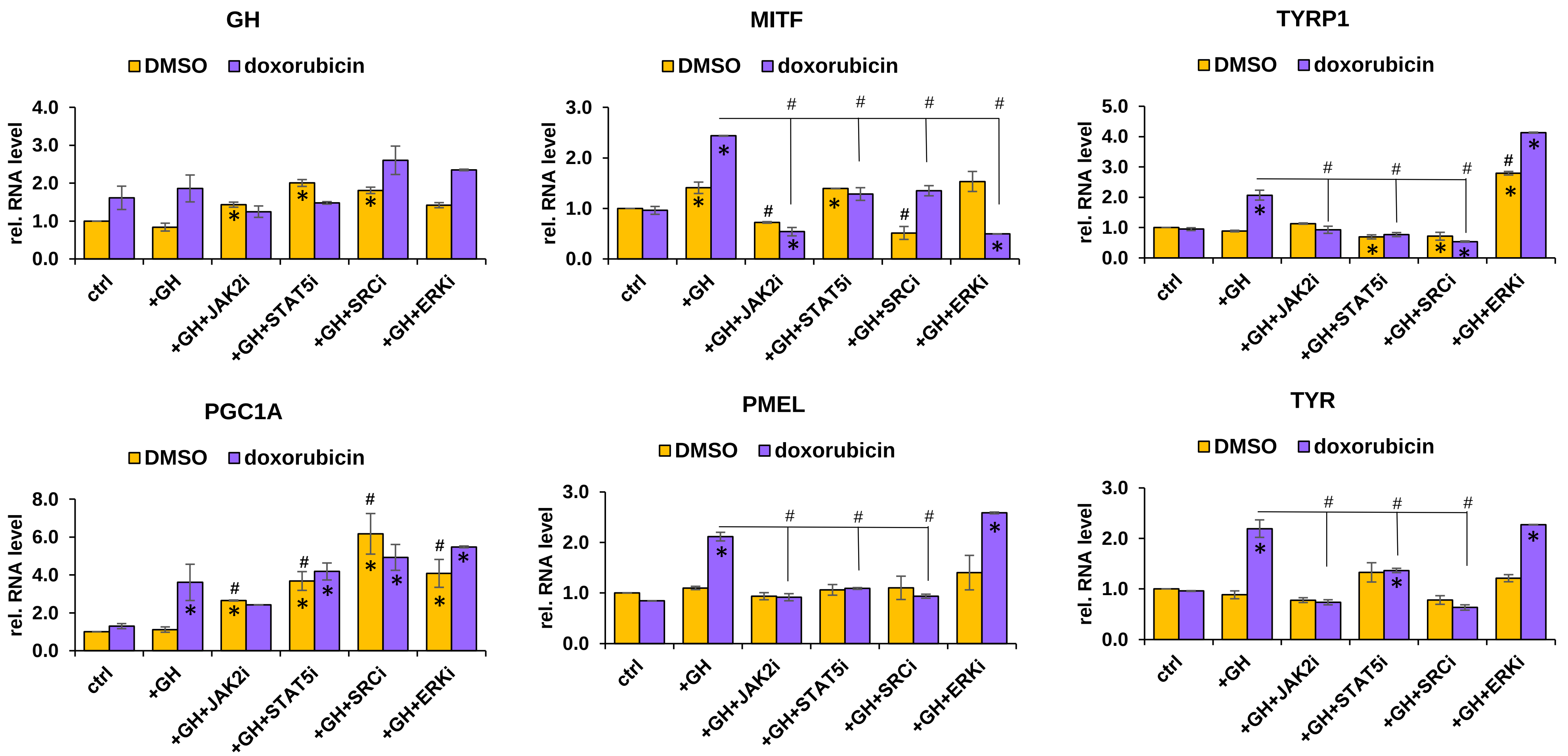

Sup-Fig 9: GH regulated MITF and MITF target gene regulation proceeds via JAK2-STAT5 and SRC regulated pathways: Human melanoma cell MDA-MB-435 (Fig 8), SK-MEL-28 (here) and SK-MEL-30 (Sup-Fig 10) were treated with /without doxorubicin in presence of GH as well as different intracellular signaling pathway inhibitors. After 24-hr treatments, RNA expression for target genes (GH, MITF and MITF targets) was quantified by RT-qPCR and normalized against expression of βTUB and ACTB as reference genes [#,*, $p < 0.05$, Wilcoxon sign rank test, n = 3; * *indicates comparison against corresponding -GH controls while # indicates comparison against corresponding +GH controls in DMSO and doxorubicin treated groups*].

Sup-Fig 10: GH regulated MITF and MITF target gene regulation proceeds via JAK2-STAT5 and SRC regulated pathways → SK-MEL-30

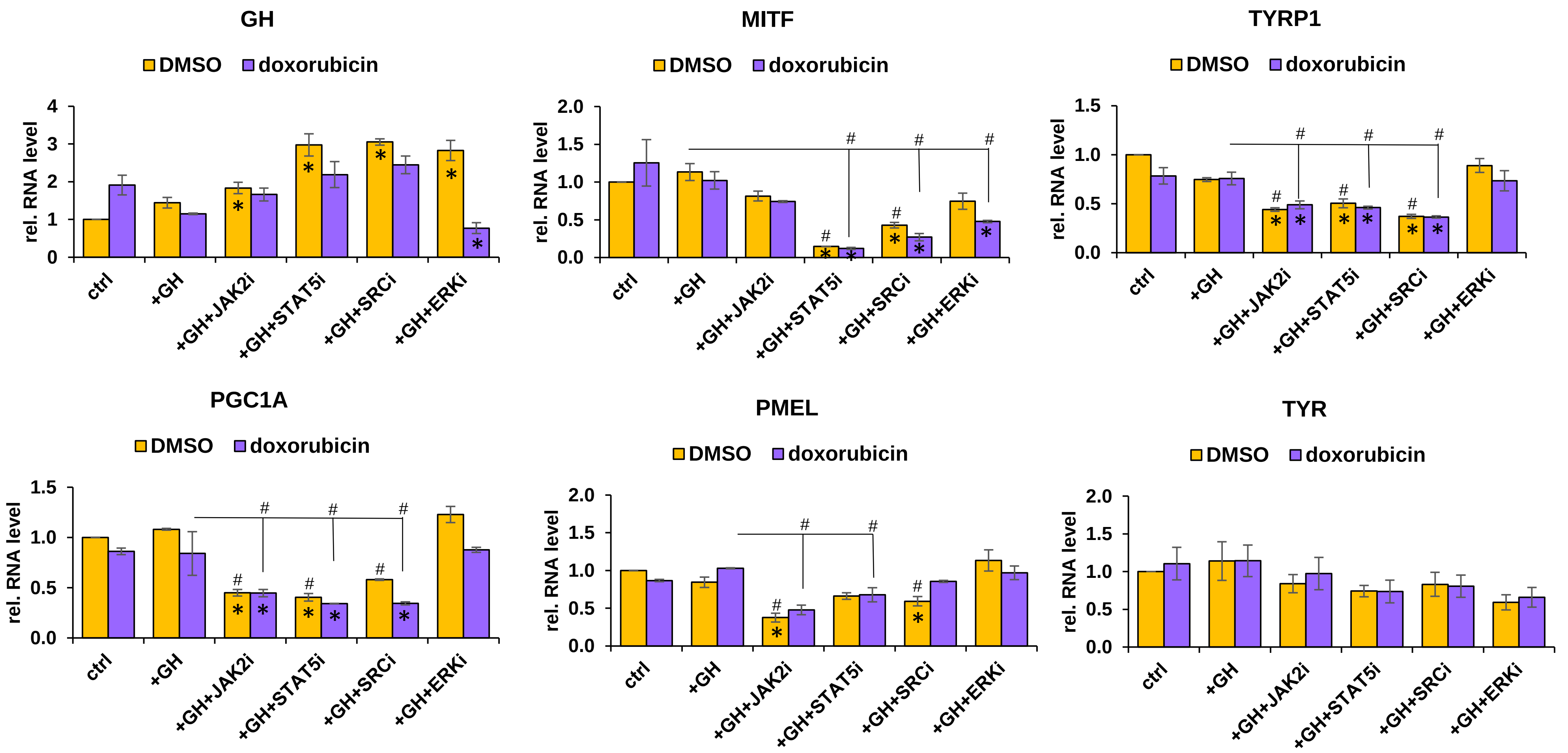

Sup-Fig 10: GH regulated MITF and MITF target gene regulation proceeds via JAK2-STAT5 and SRC regulated pathways: Human melanoma cell MDA-MB-435 (Fig 8), SK-MEL-28 (Sup-Fig 9) and SK-MEL-30 (here) were treated with /without doxorubicin in presence of GH as well as different intracellular signaling pathway inhibitors. After 24-hr treatments, RNA expression for target genes (GH, MITF and MITF targets) was quantified by RT-qPCR and normalized against expression of βTUB and ACTB as reference genes [#,*, $p < 0.05$, Wilcoxon sign rank test, n = 3; * *indicates comparison against corresponding -GH controls while # indicates comparison against corresponding +GH controls in DMSO and doxorubicin treated groups*].

Sup-Fig 11: Effect of prolactin (PRL) treatment on human melanoma cells

RT-qPCR Ct values

	MDAMB435	SKMEL28	SKMEL30	MALME3M
**GHR**	26.1	25.4	26.1	25.1
**PRLR**	30.6	29.4	30.3	30.8
**GAPDH**	15.1	15.6	14.9	15.5

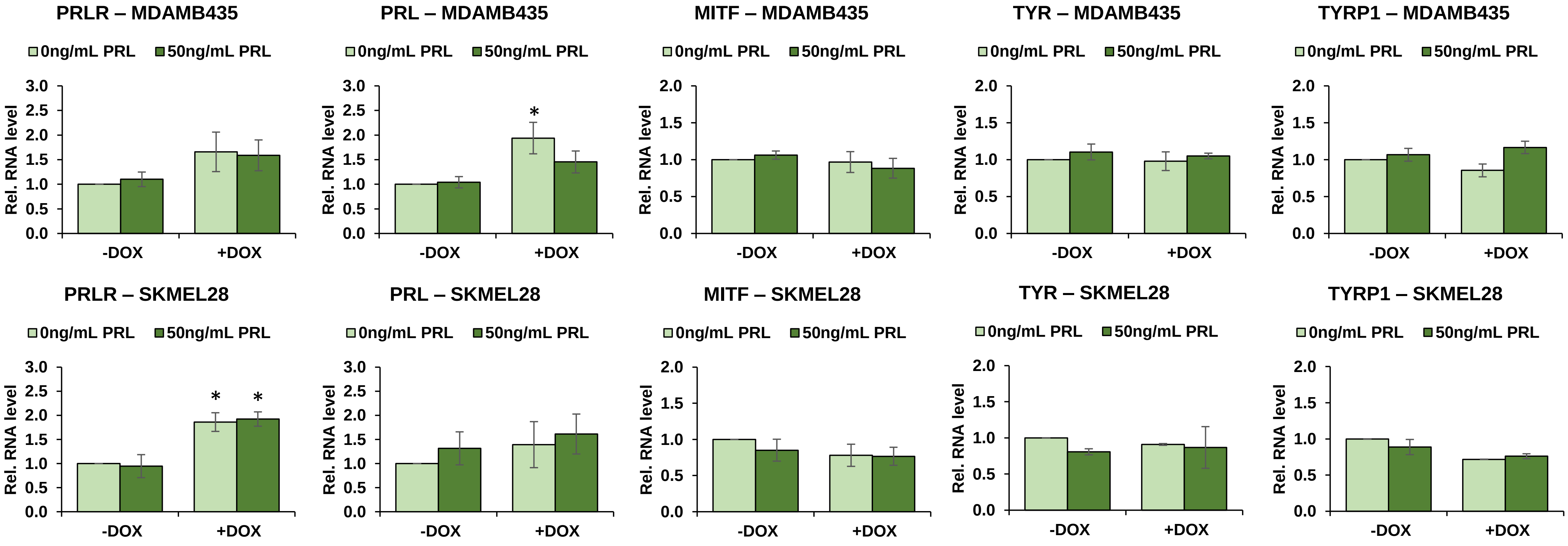

Sup-Fig 12: siRNA-mediated GHR knock down (GHRKD) in human melanoma cells:

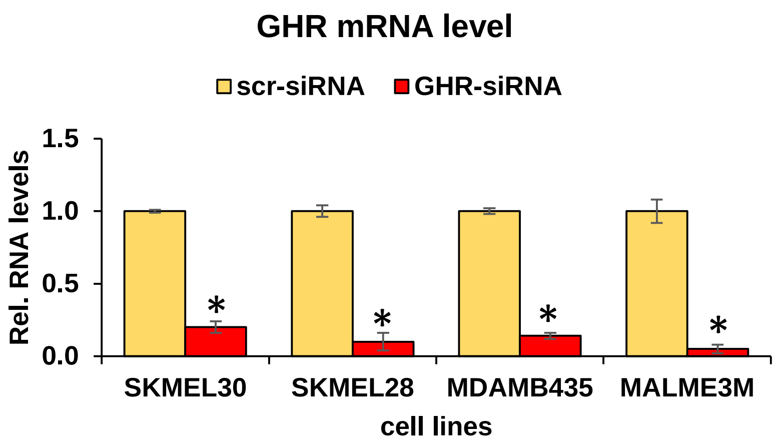

Primer List

target gene	primer direction	sequence (5'-3')
GAPDH	F	CTTTTGCGTCGCCAG
	R	TTGATGGCAACAATATCCAC
GHR	F	CTCCTCAAGGAAGGAAAATTAG
	R	GTGGAATTCGGGTTTATAGC
MITF	F	CAGTACCTTTCTACCACTTTAG
	R	CCTCTTTTTCACAGTTGGAG
PRL	F	GGTTCATCCTGAAACCAAAG
	R	CTTCAGGAGCTTGAGATAATTG
TYRP1	F	GATTCCACTCTAATAAGCCC
	R	CTGTTACAAAGTGTTCCCAG
EGFR	F	GGAAAAGAAAGTTTGCCAAG
	R	ATGAGGACATAACCAGCC
MET	F	CATGTGAATTTTCTCCTGGAC
	R	ATCTTCTTCCCAGTGATAACC
UCP1	F	ACAGCACCTAGTTTAGGAAG
	R	CTGTACGCATTATAAGTCCC
PPARGC1A	F	GCAGACCTAGATTCAAACTC
	R	CATCCCTCTGTCATCCTC
ABCA1	F	CTGGGATATGTGCAATTACG
	R	CCATACAGCAAAAGTAGAAGG
ABCA6	F	CCATATGCTATGGGAATCATC
	R	AGCTGAGAAATCTTCTTTCC
ABCA5	F	TGTTCAAAATCATGTGAGGC
	R	TTCAACTGTATAATGGCAGC
ABCC6	F	AGGCTGATTGGATCATAGTG
	R	GTTTCTCCTTCTCCTCTATCTC
ABCC8	F	GGTTTACTTTGTCTCATCCC
	R	TCTGTATTGCTCCTCTCAAG
ABCC3	F	TTTTCTGGTGGTTCACAAAG
	R	GGATCTGTCCTCTTCCTTTAG
ABCC4	F	ATGGAGATAGGAATATCGTGC
	R	TCCTCAGTGATGAGAACAAC
ACTB	F	GACGACATGGAGAAAATCTG
	R	ATGATCTGGGTCATCTTCTC
GAPDH	F	CTTTTGCGTCGCCAG
	R	TTGATGGCAACAATATCCAC
TYR	F	CAACAGCCATCAGTCTTTATG
	R	CCTTCCAGTGTATTTCTAAAGC
BCL2	F	GATTGTGGCCTTCTTTGAG
	R	GTTCCACAAAGGCATCC
BRCA1	F	CTATCATCCAAAGTATGGGC
	R	TTTCCAAGGAAGGATTTTCG
CDKN1A	F	CAGCATGACAGATTTCTACC
	R	CAGGGTATGTACATGAGGAG
DCT	F	TAGCTTGGATGACTACAACC
	R	TTCCTGAAACTGAAGGTAGAG
MLANA	F	AGCCTTGATGGATAAAAGTC
	R	CGATGATCAAACCCTTCTTG
HIF1A	F	GAAACTACTAGTGCCACATC
	R	GGAACTGTAGTTCTTTGACTC
ABCD1	F	CTCAACATCAGGGTGGAG
	R	CTCTGCGGGATGTAGAAC
PMEL	F	TAGAGAGCTACCTATCCCTG
	R	GAACCTGTAATACTTTCCGTAG
UCP1	F	ACAGCACCTAGTTTAGGAAG
	R	CTGTACGCATTATAAGTCCC
ABCA9	F	CCCAGCTTATACATTTGGAC
	R	ACCAACATGAAAAGAGTAGC
ABCA8	F	TCATTATGGCCCTTTTCTTG
	R	TTAAGAAAGCCAAAGCTACC

target gene	primer direction	sequence (5'-3')
Actb	F	GATGTATGAAGGCTTTGGTC
	R	TGTCGACTTTTATTGGTCTC
Abcc2	F	CGTATATAAGAAGGCACTAACC
	R	CAATCTGTAAAACACTGGACC
Abcc1	F	GTCTATCGTAAGGCTCTTTTG
	R	GACCAGATCATGTTAATGTACG
Abcg2	F	AAGAGCCAGTCTATGTTACC
	R	AAACTCCAGCTCTATTTTGC
Abcb8	F	GCTCTAAAGCAGAAGAACTG
	R	CCAAGACCATACAGTTGAAAG
Abcc4	F	AAACAAAGTCATCCTGTTCG
	R	CAGAAAGTTCTTGATCCTCC
Gh	F	TCCAGTCTGTTTTCTAATGC
	R	TCGAACTCTTTGTAGGTGTC
Ghr	F	ACTGTCCAGTGTACTCATTG
	R	CTGGATATCTTCTTCACATGC
Igf1	F	GACAAACAAGAAAACGAAGC
	R	ATTTGGTAGGTGTTTCGATG
Abcb5	F	CATCGGAACTATTCTTGCTG
	R	ACATTCAGGTACAAATCCAG
Tubb5	F	CTTGTTCGGTACCTACATTG
	R	CATGTTCATCGCTTATCACC
Bcl2	F	ATGACTGAGTACCTGAACC
	R	ATATAGTTCCACAAAGGCATC
Brca1	F	TCTAACCTTGGAATCGTGAG
	R	GAGTCTAGTTCAATGTAGACAG
Cdkn1a	F	CTGACAGATTTCTATCACTCC
	R	TTAAGACACACAGAGTGAGG
Dct	F	TTGATCTGTCAGAGGAAGAAG
	R	TGTATTGAAGAAAAGCCAGC
Hif1a	F	CTGATCATCTGACCAAAACTC
	R	CGTGCTGAATAATACCACTTAC
Tyr	F	AGCAGATGTGGAATTTTGTC
	R	AAATCCTTCCAGTGTGTTTC
Mlana	F	AGAACCTTGATGGACAAAAG
	R	TTCTCTTGAGAAGACAGTCG
Pmel	F	CATTAGCCCTCTACTGGATG
	R	CTTTCAATACCCTGGACAATG
Mitf	F	GCTAAAGAGAGGCAGAAAAAG
	R	GCATGTCTGGATCATTTGAC
Ucp1	F	CTTTTTCAAAGGGTTTGTGG
	R	CTTATGTGGTACAATCCACTG
Abca9	F	CTTTCCTCATGAGTGTGTTG
	R	GTCCAAATGTATAAGCTGGG
